# Using estimated probability of pre-diagnosis behavior as a predictor of cancer survival time: an example in esophageal cancer

**DOI:** 10.1186/s12874-020-00957-5

**Published:** 2020-04-03

**Authors:** Paul P. Fahey, Andrew Page, Glenn Stone, Thomas Astell-Burt

**Affiliations:** 1 School of Health Sciences, Western Sydney University, Locked Bag 1797, Penrith, NSW 2751 Australia; 2 Translational Health Research Institute, Western Sydney University, Locked Bag 1797, Penrith, NSW 2751 Australia; 3 School of Computer, Data and Mathematical Sciences, Western Sydney University, Locked Bag 1797, Penrith, NSW 2751 Australia; 4 Population Wellbeing and Environment Research Lab (PowerLab), School of Health and Society, Faculty of Social Sciences, University of Wollongong, Wollongong, NSW 2522 Australia

**Keywords:** Esophageal cancer, Survival, Health behavior, Tobacco, Alcohol, Physical activity

## Abstract

**Background:**

Information on the associations between pre-diagnosis health behavior and post-diagnosis survival time in esophageal cancer could assist in planning health services but can be difficult to obtain using established study designs. We postulated that, with a large data set, using estimated probability for a behavior as a predictor of survival times could provide useful insight as to the impact of actual behavior.

**Methods:**

Data from a national health survey and logistic regression were used to calculate the probability of selected health behaviors from participant’s demographic characteristics for each esophageal cancer case within a large cancer registry data base. The associations between survival time and the probability of the health behaviors were investigated using Cox regression.

**Results:**

Observed associations include: a 0.1 increase in the probability of smoking 1 year prior to diagnosis was detrimental to survival (Hazard Ratio (HR) 1.21, 95% CI 1.19,1.23); a 0.1 increase in the probability of hazardous alcohol consumption 10 years prior to diagnosis was associated with decreased survival in squamous cell cancer (HR 1.29, 95% CI 1.07, 1.56) but not adenocarcinoma (HR 1.08, 95% CI 0.94,1.25); a 0.1 increase in the probability of physical activity outside the workplace is protective (HR 0.83, 95% CI 0.81,0.84).

**Conclusions:**

We conclude that probability for health behavior estimated from demographic characteristics can provide an initial assessment of the association between pre-diagnosis health behavior and post-diagnosis health outcomes, allowing some sharing of information across otherwise unrelated data collections.

## Background

With an incidence of 9.3/100,000 males and 3.5/100,000 females per year, esophageal cancer led to more than half a million deaths worldwide in 2018 [1]. The majority of these deaths arise from modifiable lifestyle factors. In the US in 2014 it was estimated that 71% of male and 59% of female esophageal cancer deaths arose from modifiable lifestyle factors and that cigarette smoking, alcohol consumption and excess body weight could account for up to 50, 17 and 27% of deaths respectively [2].

While there is considerable documentation of associations between health behavior and onset of esophageal cancer [3], the impact of health behavior on survival times is less well understood [4]. A more thorough understanding of predictors of survival time is needed to assist in anticipating health service needs and for health services planning.

Health behavior prior to a cancer diagnosis is often different from health behavior post-diagnosis. Behavior prior to diagnosis can be influenced by public health activity but post-diagnosis behavior is strongly influenced by the diagnosis itself [5] and by treatment [6, 7]. As esophageal cancer has relatively short survival times (in the US, just 19% of cases survive 5-years [8]), pre-diagnosis behavior could have a strong carry over effect on survival time.

Unfortunately, investigating the effect of pre-diagnosis behavior on post-diagnosis survival can be difficult and expensive. As the disease is relatively rare, a prospective cohort study would be inefficient (on the figures above, surveillance of 100,000 men for 10 years would be expected to yield just 93 new esophageal cancer cases). Retrospective studies which enroll newly diagnosed cancer patients and ask them to recall their prior health behavior still involve considerable expense and are fraught with recall and survivor biases. In one example, an Australian study enrolling newly diagnosed esophageal cancer patients reported that patients with late-stage disease were difficult to enroll and under-represented [9].

Secondary analyses of already existing data can provide alternate, cost-effective opportunities. It is now common for governments to sponsor both regular health behavior surveys and mandatory cancer registries. For those cancer cases who contributed to a survey prior to diagnosis, their health behavior and cancer outcomes can be linked to produce a retrospective cohort. Data linkage avoids recall and survivor biases and is cost efficient (as the required data are already collected, compiled and cleaned).

But data linkage may not be feasible either. Confidentiality is one issue. But more fundamentally, as esophageal cancer is relatively rare, the number of cancer cases who happened to have previously participated in the health survey is likely to be very small. If data linkage cannot be applied, is there any other way in which these rich (and expensive) data sets can be used to help provide insights into the association between pre-diagnosis behavior and post-diagnosis survival times?

Often the only measures in common between cancer registries and national health surveys are the demographic characteristics of participants. It is known that demographically similar people are more likely to display similar health behavior than people from different demographic groups [10]. That is, different demographic groups have a different likelihood for particular behaviors. Probability of behavior calculated from demographic variables, may be a weak indicator of actual behavior, but with large data sets even weak signals are detectable.

This study investigated whether or not useful information on the association between pre-diagnosis health behaviors and post-diagnosis survival times could be obtained by analyzing cancer cases estimated probability of engaging in these behaviors. The analyses used US data and focused mainly on the three modifiable lifestyle factors identified above: cigarette smoking, alcohol consumption and excess body weight.

## Methods

### The data sets

Unit record data on esophageal cancer cases and their outcomes was extracted from the Surveillance, Epidemiology, and End Results Program (SEER) cancer registry [11]. The SEER system is administered by the National Cancer Institute. SEER currently compiles data from cancer registries covering about 28% of the US population across 13 States. Most cancers, including esophageal cancers, are recorded. De-identified unit record data made available for research include demographic measures, medical details of the cancer, treatment and outcomes (including survival time). 95.1% of esophageal cases had positive histology with just 0.4% clinical diagnosis only; the remainder having unknown (2.4%) or other confirmation methods.

Data on health behavior was extracted from the Behavioral Risk Factor Surveillance System (BRFSS) health survey [12]. The BRFSS is an annual national survey of health. It commenced in 1984 and now collects data from more than 400,000 telephone interviews each year covering adult residents of all US States and three Territories. The de-identified unit record information made available for research included demographic and health behavior measures, and State population sampling weights.

Both collections provided access to cleaned, de-identified unit record data at no cost to the researcher. Although both data collections are large, with less than 0.2% of American adults participating in BRFSS and around 4000 esophageal cancer cases being recorded in the SEER data set each year, we could only expect about eight new esophageal cancer cases each year to have participated in the previous BRFSS survey.

### Inclusions and exclusions

This analysis focusses on the 15-year period from 2001 to 2015. Data prior to 2001 are excluded due to changes in the definitions of some health behaviors variables and because earlier data may be less relevant to current behavior and outcomes. 2015 was the most recent year of SEER cancer registry data.

As esophageal cancer is rare in young ages, all cancer cases who were less than 35 years of age are excluded as being atypical. Two hundred one of 57,025 (0.3%) cases are excluded. For the BRFSS health survey, all data records from respondents 25 or more years of age who lived in one of the 13 US States represented in the SEER cancer registries are included. Including the younger respondents allows information on health behavior up to 10 years prior to cancer diagnosis to be retained.

### Outcome variable

The outcome of interest is post-diagnosis survival time in months as recorded in the SEER cancer registry data set. That is, all cases with survival less than 30.4 days after diagnosis (including cancers detected post-mortem) have a survival time of 0 months, those who died between 30.4 and 60.8 days have a survival time of 1 month, etc. The maximum possible survival time is 179 months. For those who are still alive and those who are lost to follow-up, survival time is censored at the date of last follow-up.

### Health behavior variables

The research focused mainly on measures relating to cigarette smoking, alcohol consumption and excess body weight. The choice of variables was restricted to measures available through the BRFSS health survey. The following variables, all recording self-reported behavior, were included:

- Current smoker (yes/no) which includes those who smoke daily or less than daily;
- Alcohol - heavy drinking (yes or no), which is defined as more than two standard drinks per day for men and more than one standard drink per day for women in the month prior to survey;
- Alcohol - binge drinking (yes or no), which is defined as males reporting having five or more standard drinks or females reporting 4 or more standard drinks on one occasion in the month prior to survey;
- Current smoking and alcohol consumption (yes/no), which is defined as both current smoker and an average consumption of ≥1 standard drink of alcohol per day in the past month.
- Obese (yes/no) which is BMI ≥ 30 kg/m^2^
- Undertook physical activity or exercise in the past 30 days other than regular job (yes or no)

### Demographic variables

As the cancer registry data did not include information on pre-diagnosis health behavior we estimated the probability of each pre-diagnosis health behavior for each cancer case using the available demographic variables.

Of the variables in common between the SEER cancer registry and the BRFSS health surveys we hypothesized that year, age, sex, race, marital status and State of residence could be helpful for predicting health behavior. For example, race is known to be associated with smoking [13] and alcohol dependence [14] in the US. Also, living as married ameliorates social isolation and social isolation is associated with adverse health behaviors such as smoking, higher BMI, and lower desire for exercise [15].

As age was recorded in 5-year age groups in the SEER cancer registry data, we applied the same categories to the BRFSS health survey data. Race was categorized as White; Black; Asian or Pacific Islander; and American Indian or Alaskan native. Participants in the BRFSS health survey who self-reported as mixed race (*n* = 44,670, 3.1% of total) were omitted as there was no corresponding code in the SEER cancer registry data set. Marital status was categorized as married or living as married; divorced or separated; widowed; and single.

### Other factors considered

Post-diagnosis survival time is sensitive to a range of factors, some of which could potentially confound associations with pre-diagnosis health behavior and survival time. For example, the association between health behaviors and incidence of esophageal cancer is known to differ by histological type [3, 16] and these differences appear to carry over into survival time [17, 18]. Therefore, we have conducted sub-group analyses for squamous cell carcinoma (ESCC) and adenocarcinoma (EAC). Also age is associated with survival time [19] and health behavior can change with age. Age, recorded in 5-year age groups but treated as a continuous variable, is included in the final models as a potential confounder.

Somewhat more difficult was how to address cancer stage. Cancer stage at diagnosis is an important predictor of survival time [19] and could perhaps be associated with health behavior, although this association may be an intermediary step between health behavior and survival time rather than a true confounder. For completeness we opted to adjust for cancer stage in our models. Disease stage at diagnosis (clinical assessment) was coded by SEER according to the according to the AJCC Cancer Staging Manual 6th Edition [20].

Recording of cancer stage at diagnosis was incomplete in the SEER cancer registry data; being unavailable from 2001 to 2003 and having 18% missing data across the other years. We have excluded cancer stage prior to 2004 and categorized it into 5 categories (stage I, stage II, stage III, stage IV, not specified) from 2004 onwards.

Other potential confounders of the association between behavior and survival were considered to be of lesser impact or potentially on the disease pathway. For example, while the relationship between smoking history and post-diagnosis survival may differ by gender, the effect may be small. In contrast, the choice between curative or palliative treatment is a strong predictor of survival time but may partially lie on the association pathway. (Smoking, for example, may lead to a higher probability of significant co-morbidities and these in turn influence the decision of curative treatment and, hence, survival time.) Adjustment for variables on the association pathway may remove some of the true association between health behavior and survival time.

### Eligible data records

Fifty-six thousand eight hundred twenty-four SEER esophageal cancer cases and 1,450,775 BRFSS health survey respondents met the eligibility criteria. Additional file 1 summarizes the characteristics of the two samples. Among the cancer cases, median time till death was 7 months with median follow-up time of censored observations (18.6%) was 30 months. 52.9% of cases were EAC and 33.7% ESCC. 16.1% of the BRFSS respondents were current smokers and 4.8% were judged to be heavy drinkers of alcohol. The BRFSS respondents included higher proportions of younger people and females than the SEER cases.

### Statistical analysis

The characteristics of eligible cancer registry cases and health survey respondents are summarized using counts and percentages, with the exception of survival time which is summarized using medians, quartiles and maximums.

The main analysis involves three discrete steps. Firstly, the probability of engaging in each health behavior were estimated from the BRFSS health survey data using logistic models; with a separate model for each behavior. Each modelled the probability of having the behavior of interest based on year of survey, age, sex, race, marital status and State of residence. We also allowed for differences in the probability of health behaviors between sexes and between marital statuses at different ages by including age by sex, age by marital status and marital status by sex interaction terms in each logistic model.

For example, if we let *i* represent an eligible individual from the BRFSS data set and $\hat{p_i(smoker)}$ represent the estimated probability that person *i* is a smoker, then the logistic model has the form

1 $$ logit\left(\hat{p_i(smoker)}\right)={\boldsymbol{x}}_{\boldsymbol{i}}\hat{\boldsymbol{\beta}} $$

where

$$ {\boldsymbol{x}}_{\boldsymbol{i}}\hat{\boldsymbol{\beta}}=\hat{\beta_0}+\hat{\beta_1}\left({year}_i\right)+\hat{\beta_2}\left({age}_i\right)+\hat{\beta_3}\left({sex}_i\right)+\hat{\beta_{4-6}}\left({race}_i\right)+\hat{\beta_7}\left( marital\ {status}_i\right)+\hat{\beta_{8-19}}\left( State\ of\ {residence}_i\right)+\hat{\beta_{20}}\left({age}_i\right)\left({sex}_i\right)+\hat{\beta_{21}}\left({age}_i\right)\left( marital\ {status}_i\right)+\hat{\beta_{22}}\left({sex}_i\right)\left( marital\ {status}_i\right) $$

and the $\hat{\beta}$ ’s quantify the relationships between the demographic characteristics of the respondents and their likelihood of smoking.

To correct for the complexities in the BRFSS health survey sampling and non-response we weighted the logistic models by the sampling weights provided. In 2011, the BRFSS introduced a new method of calculating sampling weights which improved the weighting of some variables including race and marital status. However, as both systems weight to the State totals, we do not differentiate between the different type of weights in this analysis. We excluded data records with extreme sampling weights: those which fell in either the top or bottom 0.5% of the distribution. To assist the models to converge we use Firth’s bias reduced penalized-likelihood when fitting the models; using the logistf package (version 1.23) in R software (version 3.5.2). The fitted models are summarized in Additional file 5.

Year and age category were fitted as numeric variables while sex, race, marital status and State of residence are categorical. Preliminary investigations (not reported) confirmed that a linear model was reasonable for both year and age category. Year is coded as 0 for 2001 through to 14 for 2015 for analysis.

We confirmed that the chosen risk profiling variables were indeed predictors of each health behavior by visual inspection of odds ratios from logistic regression models. To help gauge the predictive ability of each demographic variable we present areas under the curve (AUC) of the receiver operating characteristic (ROC) curve for each predictor alone and for the full logistic model using the pROC package (version 1.13.0) in R software. The higher above 0.5 the AUC, the greater the ability of the model to predict the health behavior.

In the second step of the analysis, for each esophageal cancer case in the SEER cancer registry, we estimated their probability of participating in each health behavior by substituting their demographic characteristics into the logistic predictive model for that behavior.

For example, if we let *j* represent an eligible cancer case from the SEER data set and ***x***_***j***_ the set of observed values of the demographic variables for individual *j* and $\hat{\boldsymbol{\beta}}$ represent the regression coefficients for the model predicting smoking (eq. 1 above), then we estimated the probability of cancer case *j* being a smoker as

2 $$ \hat{p_j(smoker)}=\frac{e^{{\boldsymbol{x}}_{\boldsymbol{j}}\hat{\boldsymbol{\beta}}}}{1+{e}^{{\boldsymbol{x}}_{\boldsymbol{j}}\hat{\boldsymbol{\beta}}}} $$

As we were specifically interested in health behavior prior to diagnosis we trialed three pre-diagnosis time points: 1, 5 and 10 years prior to diagnosis. This entailed substituting diagnosis year minus 1, 5 or 10 as the year variable of the logistic model and 5-age group minus 0, 1 or 2. To avoid extrapolating earlier than the observed data, the 5-year lag analysis was restricted to esophageal cancer cases from 2006 to 2015 and the 10-year lag model was restricted to cases from 2011 to 2015.

In the third step of the analysis, the relationship between the estimated probability of each behavior and survival was investigated using Cox regression models using the survival package (version 2.43–3) in R software. Separate models were fitted for each behavior. Results are presented as hazard ratios (HRs) with associated 95% confidence intervals (CIs) and *p*-values. Models were fitted with and without correction for age and cancer stage at diagnosis.

For example, the Cox model of survival time of cancer case *j* relative to their estimated probability of smoking, adjusting for age and disease stage, could be written

3 $$ S\left(t,x,\beta \right)={\left[{S}_0(t)\right]}^{\mathit{\exp}\left({\beta}_1^{\ast}\left(\hat{p_j(smoker)}\right)+{\beta}_2^{\ast}\left({age}_j\right)+{\beta}_3^{\ast}\left( cancer\ {stage}_j\right)\right)} $$

where $\hat{p_j(smoker)}$, a number between 0 and 1, is the estimated probability that the SEER cancer case is a smoker from Eq. (2). The * superscript is just to highlight that these *β* ’s are different to the *β* ’s listed in Eq. (1). Under this model ${e}^{\beta_1^{\ast }}$ is the hazard ratio for the estimated probability of smoking, adjusted for age and disease stage.

Subgroup analyses were performed for ESCC and EAC histological types. Missing values were excluded from analysis.

## Results

Each of the risk profile variables were related to each of the health behaviors [see Additional file 2]. For example, the prevalence of smoking decreased over the study period (odds ratio (OR) = 0.98, 95% confidence interval (CI) 0.98–0.98 for each later year); the prevalence of obesity increased over time (OR = 1.03, 95% CI 1.03–1.03 for each additional year); each 5-year increase in age is associated with decreasing prevalence of smoking (OR = 0.90, 95% CI 0.90–0.90) and decreased risk of binge drinking (OR = 0.82, 95% CI 0.82–0.82); females have lower prevalence of smoking (OR = 0.74, 95% CI 0.74,0.74); when compared to those who are married, people who are single have higher prevalence of daily smoking (OR = 2.14, 95% CI 2.14–2.14), risk of binge drinking (OR = 1.90, 95% CI 0.90–0.90) and risk of concurrently smoking and regular drinking (OR = 2.50, 95% CI 2.50–2.50); people classifying as American Indian or Alaskan Native have higher prevalence of daily smoking (OR = 1.69, 95% CI 1.68–1.69) and people classified as black have higher risk of obesity (OR = 1.75, 95% CI 1.75–1.75) than those who are classified as white; residents of Kentucky are more likely to smoke (OR = 2.50, 95% CI 2.49–2.50) residents of Utah are less likely to be heavy drinkers (OR = 0.52, 95% CI 0.52–0.52) than Californians.

Of the fitted logistic models, the model predicting binge drinking (AUC 0.74) appeared most accurate and the model for predicting obesity (AUC 0.59) appeared least accurate.

Table 1 shows the associations between post-diagnosis survival time and probability of each pre-diagnosis health behavior. Each line presents results from separate Cox regression models; for each health behavior. The columns present results from three separate models: the unadjusted model with the probability of behavior 1 year prior to diagnosis as the only predictor; the one-year lag model adjusted for age and cancer stage at diagnosis; and the adjusted model with a 10-year lag. The hazard ratios reported show the impact of a 0.1 increase in the probability of participating in that behavior. Tables 2 and 3 provide the same results for the ESCC and EAC histological types separately. Both adjusted variables (age and cancer stage at diagnosis) are significant predictors of survival [see Additional file 3]. Result for the 5-year lag model [see Additional file 4] are similar to corresponding one-year lag models shown.

**Table 1:** Association Between Survival Time and Probability of Pre-Diagnosis Health Behavior; All Esophageal Cancers

	1 year lag, unadjusted			1 year lag, adjusted^b^			10 year lag, adjusted^b^		
Health behavior	HR^a^	95% CI	*P* value	HR^a^	95% CI	*P* value	HR^a^	95% CI	*P* value
Current smoker	0.99	0.99,1.01	0.252	1.20	1.18,1.22	< 0.001	1.18	1.15,1.21	< 0.001
Alcohol - Heavy drinking	0.53	0.50,0.56	< 0.001	0.82	0.76,0.88	< 0.001	1.16	1.04,1.30	0.011
Alcohol - Binge drinking	0.76	0.74,0.77	< 0.001	0.96	0.93,0.99	0.012	1.04	0.99,1.08	0.093
Current smoking and alcohol consumption	0.87	0.83,0.92	< 0.001	1.93	1.79,2.07	< 0.001	1.69	1.56,1.84	< 0.001
Undertook exercise in past 30 days other than regular job	0.78	0.77,0.79	< 0.001	0.82	0.81,0.84	< 0.001	0.80	0.78,0.83	< 0.001
Obese	0.95	0.94,0.97	< 0.001	1.04	1.03,1.06	< 0.001	1.10	1.07,1.14	< 0.001

Abbreviations: *CI* Confidence interval, *HR* Hazard ratio

^a^The hazard ratio describes the impact of a 0.1 increase in the probability of having the specified health behavior

^b^Adjusted for age and cancer stage at diagnosis

**Table 2:** Association Between Survival Time and Probability of Pre-Diagnosis Health Behavior; Esophageal Squamous Cell Carcinomas

Health behavior	1 year lag, unadjusted			1 year lag, adjusted^b^			10 year lag, adjusted^b^		
	HR^a^	95% CI	P value	HR^a^	95% CI	*P* value	HR^a^	95% CI	*P* value
Current smoker	0.99	0.97,1.01	0.215	1.20	1.17,1.23	< 0.001	1.19	1.16,1.22	< 0.001
Alcohol - Heavy drinking	0.50	0.45,0.56	< 0.001	0.78	0.69,0.88	< 0.001	1.30	1.08,1.57	0.007
Alcohol - Binge drinking	0.75	0.73,0.78	< 0.001	0.95	0.90,1.00	0.035	1.09	1.02,1.17	0.013
Current smoker and ≥ 1 alcoholic drink /day	0.85	0.79,0.93	< 0.001	1.93	1.72,2.16	< 0.001	1.68	1.51,1.88	< 0.001
Did exercise in past 30 days other than regular job	0.78	0.76,0.79	< 0.001	0.82	0.80,0.85	< 0.001	0.79	0.77,0.82	< 0.001
Obese	0.97	0.94,0.99	0.004	1.07	1.04,1.10	< 0.001	1.08	1.04,1.11	< 0.001

Abbreviations: *CI* Confidence interval, *HR* Hazard ratio

^a^The hazard ratio describes the impact of a 0.1 increase in the probability of having the specified health behavior

^b^Adjusted for age and cancer stage at diagnosis

**Table 3:** Association Between Survival Time and Probability of Pre-Diagnosis Health Behavior; Esophageal Adenocarcinomas

	1 year lag, unadjusted			1 year lag, adjusted^b^			10 year lag, adjusted^b^		
Health behavior	HR^a^	95% CI	P value	HR^a^	95% CI	*P* value	HR^a^	95% CI	*P* value
Current smoker	1.00	0.98,1.01	0.625	1.20	1.18,1.23	< 0.001	1.18	1.16,1.21	< 0.001
Alcohol - Heavy drinking	0.55	0.51,0.59	< 0.001	0.85	0.77,0.93	< 0.001	1.10	0.95,1.26	0.216
Alcohol - Binge drinking	0.76	0.74,0.78	< 0.001	0.97	0.93,1.01	0.121	1.01	0.96,1.07	0.722
Current smoker and ≥ 1 alcoholic drink /day	0.89	0.83,0.95	< 0.001	1.93	1.76,2.11	< 0.001	1.79	1.65,1.94	< 0.001
Did exercise in past 30 days other than regular job	0.78	0.77,0.80	< 0.001	0.82	0.81,0.84	< 0.001	0.83	0.81,0.85	< 0.001
Obese	0.94	0.92,0.96	< 0.001	1.03	1.00,1.05	0.028	1.07	1.04,1.10	< 0.001

Abbreviations: *CI* Confidence interval, *HR* Hazard ratio

^a^The hazard ratio describes the impact of a 0.1 increase in the probability of having the specified health behavior

^b^Adjusted for age and cancer stage at diagnosis

Smoking 1 year prior to diagnosis appears to be unrelated to survival until adjustment for age and disease stage at diagnosis. In the adjusted model, each 0.1 increase in the probability of pre-diagnosis smoking is associated with a 20% (HR 1.20, 95% CI 1.18–1.22) increase in post-diagnosis hazard with no discernible difference in results for ESCC and EAC subgroups.

Results for alcohol consumption are mixed. When using behavior 1 year prior to diagnosis as the predictor, a 0.1 increase in the probability of heavy drinking appears to be protective of survival even after adjustment for age and cancer stage at diagnosis (HR 0.82, 95% CI 0.76–0.88). However, when looking at behavior 10 years prior to diagnosis, the adjusted model finds heavy drinking to be detrimental to post-diagnosis survival in ESCC (HR 1.30, 95% CI 1.08–1.57) and with no discernable association in EAC (HR 1.10, 95% CI 0.95–1.26). The pattern of results for binge drinking is quite similar.

A 0.1 increase in the probability of concurrently smoking and drinking ≥1 standard drink per day in the year prior to diagnosis is associated with double the risk of death (HR = 1.93, 95% CI 1.79–2.07), after adjustment for age and cancer stage with no difference between ESCC and EAC.

After adjustment, a 0.1 increase in probability of obese 1 year prior to diagnosis is associated with an apparently trivial increase in post-diagnosis hazard (HR 1.04, 95% CI 1.03–1.06). A slightly larger hazard (HR 1.10, 95% CI 1.07–1.14) was recorded for a 0.1 increase in the probability of obese 10 years prior to diagnosis. A 0.1 increase in the probability of exercise outside employment 1 year prior to diagnosis is associated with improved survival (HR 0.82, 95% CI 0.81–0.84) with little difference between ESCC and EAC.

## Discussion

The results above appear to support of the proposition that demographic-derived estimates of the probability of health behaviors can assist in identifying association between pre-diagnosis health behavior and post-diagnosis survival time in esophageal cancer. The hazard ratios quoted in this paper show the increased hazard of death associated with each additional 0.1 probability of the health behavior of interest. That is, we are reporting the association between the estimated likelihood of engaging in a particular behavior and survival time. This is quite different from the association between the actual health behavior and survival time and more difficult to interpret. Never-the-less, there is consistency between the results of the present study and previously published results: especially in the presence and direction of associations.

We have found that a 0.1 increase in the probability of smoking 1 year prior to diagnosis, adjusted for age and cancer stage at diagnosis, had an estimated HR of 1.20 (95% CI 1.18–1.22) in esophageal cancer survival. This association is consistent with findings from previous meta analyses such as HR 1.41 (95% CI 1.22,1.64) [21] for smoking status at time of diagnosis in mainly ESCC patients and HR 1.19 (95% CI 1.04,1.36) for ever smoking [4] in ESCC (although no evidence of association in EAC). Some more recently published studies found similar statistically significant HRs including HR = 1.28 [22] and HR = 1.34 [23] both from China, and HR = 1.22 from a study across two sites in US and Canada [24]. In contrast, recent results from Japan HR = 0.97 [25] failed to find evidence of association between pre-diagnosis smoking and post-diagnosis survival time. A study from South Africa reported an unadjusted HR = 0.92 [26] but the present study has shown the importance of adjustment for confounders such as age and cancer stage at diagnosis.

The current analyses found that increased probability for ‘at risk’ alcohol consumption in the year prior to diagnosis were generally protective of survival but that a 0.1 increase in ‘at risk’ alcohol behavior 10 years prior to diagnosis was detrimental to survival in ESCC (heavy drinking HR 1.30 95% CI 1.08–1.57, binge drinking HR 1.09, 95% CI 1.02–1.17). The 10 year results are consistent with a previous meta-analysis [4] which found that ever drinking alcohol produced a significant increase in hazard (HR 1.36, 95% CI 1.15, 1.61) in ESCC but non-significant HR of 1.08 (95% CI 0.85, 1.37) in EAC although ever drinking and ‘at risk’ drinking are widely separate. More recent results from China HR = 1.58 [22], HR = 1.45 [23] and Japan HR = 2.37 (95% CI 1.24,4.53) [25] also support the detrimental impact of pre-diagnosis alcohol consumption on post-diagnosis survival.

The unexpectedly protective result for alcohol consumption one-year prior to diagnosis could indicate insufficient adjustment for confounding (such as comorbidities or health symptoms) or weaknesses in the measurement tool (such as biases in the self-reporting of alcohol consumption in standard drinks).

Previous authors have found that pre-diagnosis smoking and alcohol consumption combined produce a disproportionately high risk to post-diagnosis survival (for example, HR 3.84, 95% CI 2.02,7.32 for ESCC [17]). We have also found that a 0.1 increase in the probability of concurrent daily smoking and consuming one or more alcoholic drinks per day 1 year prior to diagnosis, adjusted for age and cancer stage at diagnosis, had a relatively high estimated HR of 1.93 (95% CI 1.79,2.07).

We observed that a 0.1 increase in the probability of obese 1 year prior to diagnosis was associated with slightly higher risk of death adjusted HR = 1.04 (95% CI 1.03,1.06) mainly associated with ESCC (HR 1.07 95% CI 1.04,1.10). The association seems small and the literature on obesity is sparse with mixed findings. One review found pre-diagnosis obesity could be associated with higher risks of death in cancer (specifically breast, prostate and colorectal cancers) [27] but a later study reported that pre-diagnostic obesity increased hazard for all cancers except cancers of the upper digestive tract (obese compared to normal weight HR 0.87, 95% CI 0.62,1.22) [28]. More recently a North American study [24] found recalled obesity in early adulthood was associated with lower survival times than normal weight (HR 1.77, 95% CI 1.25, 2.51). The measure of obesity available in this study may not be optimal.

### Exercise

We found that a 0.1 increase in probability of pre-diagnosis physical activity outside of the workplace was associated with improved survival (adjusted HR = 0.82, 95% CI 0.81,0.84). This is consistent with a recent review [29] which found the relative risk of death between the highest versus lowest category of physical activity to be 0.71 (95% CI 0.57,0.89) for esophageal cancer.

### Strengths and weaknesses

Our analyses using estimated probability for health behaviors has produced results which have some face validity. A strength of this example is that the data sets used are large, public domain and well understood. Any interested researcher can reproduce, refine and/or extend these analyses using the same data sets.

Both the data sets and the analysis technique used have some limitations and weaknesses. In relation to the data sets, there are response biases within the BRFSS [30] which the sampling weights may not have fully addressed. Further, the measures of behavior available are limited and are dictated by the existing data base which was designed for other purposes and is not optimized for our research question.

For the model, estimating the probability of a behavior is less accurate than a direct measure of behavior and conveys less information about that behavior: so will have less power for detecting associations. There may be residual confounding from unmeasured variables (such as education, socio-economic status or comorbidities). Finally, omitting interactions with year may have contributed to the apparent lack of difference in outcomes between behavior one, five and 10 years prior to diagnosis.

## Conclusion

The rarer the disease, the less feasible it is to conduct either prospective cohort studies or record linkage (retrospective cohort) studies. Retrospective data collection (including case-control studies) are fraught with recall and survivor biases. Exploiting existing data provides cost-effective opportunities for investigations but may require different methodologies.

Analyses of the associations between estimated probability for pre-diagnosis health behavior (based on demographic characteristics) and survival time in esophageal cancer produced results with some face validity. Expressing associations in units of changes in the probability of the health behavior was cumbersome. However, the required data are already available, allowing relatively quick and inexpensive investigations of possible associations between pre-diagnosis behavior and post-diagnosis outcomes for relatively rare diseases. And of course, most diseases are relatively rare.

## Supplementary information

**Additional file 1:Table S1.** Disease Characteristics and Outcomes of Eligible SEER Cancer Registry Esophagael Cancer Cases and BRFSS Health Survey Respondents 2001–2015.

**Additional file 2:Table S2.** Associations Between the Selected Demographic Variables and Health Behaviors in the BRFSS Health Survey Data Set.

**Additional file 3:Table S3.** Relationship Between Adjusted Variables and Survival Time.

**Additional file 4:Table S4.** Association Between Survival Time and 5-Year Pre-Diagnosis Health Behavior.

**Additional file 5:Table S5.** Logistic Regression Models Predicting Health Behaviors from Demographic Variables.

## Data Availability

The SEER Research Data used in this study are made available to the public at no cost, subject to data-use agreement (https://seer.cancer.gov/data/). The BRFSS data sets used in this study are freely available from https://www.cdc.gov/brfss/index.html.

## Abbreviations

AUC: Area under the curve
BMI: Body mass index
BRFSS: Behavioral Risk Factor Surveillance System
CI: Confidence interval
EAC: Esophageal adenocarcinoma
ESCC: Esophageal squamous cell carcinoma
HR: Hazard ratio
OR: Odds ratio
ROC: Receiver operating characteristic curve
SEER: Surveillance, Epidemiology, and End Results Program
